# Association between sub-phenotypes identified using latent class analysis and neurological outcomes in patients with out-of-hospital cardiac arrest in Japan

**DOI:** 10.1186/s12872-024-03975-z

**Published:** 2024-06-14

**Authors:** Hiroyuki Tamura, Hideto Yasuda, Takatoshi Oishi, Yutaro Shinzato, Shunsuke Amagasa, Masahiro Kashiura, Takashi Moriya

**Affiliations:** 1 Department of Emergency and Critical Care Medicine, Saitama Medical Center, Jichi Medical University, 1-847 Amanuma-Cho, Omiya-Ku, Saitama-Shi, Saitama, 330-8503 Japan; 2 Division of Emergency and Transport Services, National Center for Child Health and Development, Tokyo, Japan

**Keywords:** Cardiac arrest, Latent class analysis, Sub-phenotype, Glasgow coma scale, Partial pressure of arterial oxygen

## Abstract

**Background:**

In patients who experience out-of-hospital cardiac arrest (OHCA), it is important to assess the association of sub-phenotypes identified by latent class analysis (LCA) using pre-hospital prognostic factors and factors measurable immediately after hospital arrival with neurological outcomes at 30 days, which would aid in making treatment decisions.

**Methods:**

This study retrospectively analyzed data obtained from the Japanese OHCA registry between June 2014 and December 2019. The registry included a complete set of data on adult patients with OHCA, which was used in the LCA. The association between the sub-phenotypes and 30-day survival with favorable neurological outcomes was investigated. Furthermore, adjusted odds ratios (ORs) and 95% confidence intervals (CIs) were estimated by multivariate logistic regression analysis using in-hospital data as covariates.

**Results:**

A total of, 22,261 adult patients who experienced OHCA were classified into three sub-phenotypes. The factor with the highest discriminative power upon patient’s arrival was Glasgow Coma Scale followed by partial pressure of oxygen. Thirty-day survival with favorable neurological outcome as the primary outcome was evident in 66.0% participants in Group 1, 5.2% in Group 2, and 0.5% in Group 3. The 30-day survival rates were 80.6%, 11.8%, and 1.3% in groups 1, 2, and 3, respectively. Logistic regression analysis revealed that the ORs (95% CI) for 30-day survival with favorable neurological outcomes were 137.1 (99.4–192.2) for Group 1 and 4.59 (3.46–6.23) for Group 2 in comparison to Group 3. For 30-day survival, the ORs (95%CI) were 161.7 (124.2–212.1) for Group 1 and 5.78 (4.78–7.04) for Group 2, compared to Group 3.

**Conclusions:**

This study identified three sub-phenotypes based on the prognostic factors available immediately after hospital arrival that could predict neurological outcomes and be useful in determining the treatment strategy of patients experiencing OHCA upon their arrival at the hospital.

**Supplementary Information:**

The online version contains supplementary material available at 10.1186/s12872-024-03975-z.

## Background

Patients experiencing out-of-hospital cardiac arrest (OHCA) exhibit a very low resuscitation rate [1]. The prognosis of patients experiencing OHCA depends on various clinical factors, including patient factors (age, cause of cardiac arrest, and rhythm of cardiac arrest), cardiopulmonary resuscitation (CPR) factors (bystander CPR, quality of CPR, and time from cardiac arrest to CPR), and resuscitation treatment factors (adrenaline administration and airway management). Due to the high heterogeneity of patient groups [2–10], several studies have failed to show any association between these clinical factors, although they may influence the patient’s prognosis [11–18]. Improving the prognosis of patients experiencing OHCA remains a major concern.

Various factors have been reported to influence the prognosis in the management of critically ill patients in disease groups with high patient heterogeneity, such as those with sepsis and acute respiratory distress syndrome (ARDS) [19–22]. Recently, subgroup classification systems based on clinical factors and biomarkers have been proposed for disease groups, such as sepsis, ARDS, acute kidney injury, and acute pancreatitis [23–25]. Machine learning latent class analysis (LCA) using clustering techniques can classify disease groups from a single phenotype into subgroups (sub-phenotypes) that differ in characteristics from other groups. Moreover, LCA is useful in examining the differential effects of therapeutic interventions [26–31]. Identifying sub-phenotypes that are more closely associated with clinical outcomes may explicate the factors associated with prognosis, which may be more beneficial than treating a single group of diseases with high patient heterogeneity. Identification of prognosis-associated factors via discerning sub-phenotypes may lead to a better understanding of the pathogenesis of the disease, discovery of new targets for treatment, and development of more targeted therapies [23–25].

Even in OHCA, patient heterogeneity is high, and various factors may influence the prognosis. Therefore, it is important to consider not only a single factor but also the combined influence of various factors in this type of disease group to identify the population with the greatest impact on prognosis. Two studies on machine learning LCA for OHCA have been reported in Japan, one of which was classified by shockable rhythm, while the other by non-shockable rhythm [32, 33]. It is important to classify OHCA according to the initial cardiac rhythm and validate the sub-phenotypes. Initial rhythm may not be the most significant prognostic factor. This finding should be validated in LCA that includes other prognostic factors for all OHCA cases. It is crucial to make treatment decisions for patients experiencing OHCA soon after they arrive at the hospital. Therefore, it is important to identify sub-phenotypes using only pre-hospital factors and factors that can be measured immediately upon arrival at the hospital to aid in making treatment decisions.

This study was not limited to cardiac rhythm. Herein, we evaluated the association between the identified sub-phenotypes and clinical outcomes at 30 days by performing LCA using machine learning with pre-hospital prognostic factors and factors that could be measured immediately upon arrival at the hospital.

## Methods

### Study design and setting

This was a multicenter, retrospective, observational study, which used the OHCA registry maintained by the Japanese Association for Acute Medicine (JAAM). Specifically, this is a registry of patients who experienced OHCA and were transported to 91 hospitals in Japan between June 1, 2014, and December 31, 2019. This registry collects pre- and post-hospital information regarding patients who experience OHCA in Japan [34]. Pre-hospital information was collected from the All-Japan Utstein Registry of the Fire and Disaster Management Agency, the details of which were reported in 2010. Post-hospital information was collected from the medical staff, including the physicians, at each institution. All the pre- and post-hospital information was registered in a web-based system. Information regarding extraction factors was not stripped or concealed because the physicians overseeing the conduct of the study collected these data at each center, and the outcome assessors were not blinded.

### Ethical considerations

Approval for collecting JAAM-OHCA information was obtained from the ethics committee of each participating hospital. Approval for the conduct of this study (that is, secondary analysis) was obtained from the Ethics Committee of the Jichi Medical University Saitama Medical Center (approval number: S19-016). Since the patients with OHCA evaluated in the current registry-based study did not receive interventions that deviated from general CPR practices, the typical requirement for informed consent was waived by the ethics review committee of each participating institution. However, together with other institutions, we provided an opt-out procedure on the website of the Department of Emergency Medicine, Jichi Medical University Saitama Medical Center. This study was conducted in accordance with the guidelines specified in the Strengthening the Reporting of Observational Studies in Epidemiology (STROBE) statement and the tenets of the Declaration of Helsinki and its subsequent amendments [35].

### Study participants

Patients who experienced OHCA were included in this study. Patients younger than 18 years of age and those with missing factors used in the LCA were excluded.

### Data collection

The pre-hospital data collected included patient factors (age, sex, initial cardiac rhythm, and return of spontaneous circulation), circumstantial factors (witness and bystander CPR), treatment factors (pre-hospital physician intervention, defibrillation, adrenaline administration, and advanced airway management), and time factors (time from call to CPR and time to hospital arrival). Post-hospital data obtained within minutes of arrival at the hospital included Glasgow Coma Scale (GCS), body temperature, cardiac rhythm on arrival, and blood gas analysis (pH, PaO_2_ (partial pressure of oxygen in arterial blood), PaCO_2_ (partial pressure of carbon dioxide in arterial blood), bicarbonate, base excess, and lactate). Factors that required several minutes to obtain, such as creatinine levels, were excluded from the survey. The following diagnostic and treatment factors were collected after arrival at the hospital: cause of cardiopulmonary arrest (CPA), extracorporeal membrane oxygenation (ECMO)- assisted cardiopulmonary resuscitation (ECPR), intra-aortic balloon pumping (IABP), percutaneous coronary intervention (PCI), and targeted temperature management (TTM) [36–42]. Thirty-day survival and cerebral performance category (CPC) were the prognostic factors collected. Data were collected individually by the treating physician, and the outcome assessors were not blinded.

### Outcome measures

The primary outcome of this study was good neurological outcome 30 days after cardiac arrest. A good neurological outcome was defined as a CPC score of 1 or 2 [43]. These outcomes were determined based on the findings of previous studies [32, 33]. The secondary outcome was 30-day survival.

### Statistical analyses

#### Selected variables for LCA

We selected clinically important variables that could be measured immediately upon arrival at the hospital, with results available within a short time frame of 1–2 min. These variables were selected from the database, and a total of 21 variables were used as potential candidates for LCA. The covariates included in the LCA in this study were: demographic information (age and sex), pre-hospital data (presence of witnesses, bystander CPR, bystander defibrillation, pre-hospital adrenaline administration, pre-hospital advanced airway management, initial cardiac rhythm, pre-hospital physician contact, time from call to CPR, time from emergency call to hospital arrival, and return of spontaneous circulation (ROSC) at hospital admission), and post-hospital data [initial cardiac rhythm, body temperature, blood, gas analysis (pH, PaO_2_, PaCO_2_, bicarbonate, base excess, and lactate), and GCS]. Missing values without imputation were excluded, and only complete cases were included in the analysis.

### LCA/ model fitting/ evaluation of the model

We conducted LCA using the R package VarSelLCM (R Foundation for Statistical Computing, Vienna, Austria) to identify the underlying classes in the study population. LCA is a statistical technique that allows the identification of unobserved subgroups within a population based on observed categorical variables. To determine the optimal number of clinically meaningful sub-phenotypes, we employed model selection criteria, such as the Bayesian Information Criterion (BIC) and adjusted BIC. These criteria helped us assess the goodness of fit of models with different numbers of latent classes and select the most appropriate model. Cluster analysis was conducted using 2–5 classes to explore the range of potential sub-phenotypes in the population. Once the optimal number of classes was determined, the parameters of the selected model were estimated using maximum likelihood estimation. This estimation enabled us to obtain the probabilities of class membership for each individual in the dataset. To evaluate the discriminative power of the identified latent classes, the discriminative power of each variable was computed by calculating the logarithm of the ratio of the probabilities associated with the variable and its relevance to clustering. Higher variable index indicates a stronger association between the variable and the clustering process, indicating a higher discriminative power. After creating sub-phenotypes based on the model, the following analyses were performed: Continuous variables were presented as the median and interquartile range (IQR) for demographic characteristics, pre-hospital data, and post-hospital data. Categorical variables were presented as proportions and percentages. In addition, the Kruskal–Wallis rank sum test was used to test the continuous variables, whereas the Chi-square or Fisher's exact tests were used to test the categorical variables.

### Associations between sub-phenotypes and outcomes

The primary outcome was 30-day survival with favorable neurological outcome (Cerebral Performance Category 1 or 2). The secondary outcome was 30-day survival rate. The investigation of 30-day survival was based on the nationwide Utstein registry maintained by the Fire and Disaster Management Agency. Neurological outcomes were evaluated through follow-up interviews conducted by the attending physicians responsible for patient care. The association between each identified subgroup and the outcomes was initially assessed using Chi-square or Fisher's exact tests. Subsequently, logistic regression analysis was performed with treatment interventions as covariates to calculate the odds ratios (ORs) and 95% confidence intervals (CIs) for each subgroup. The final model included the following covariates: IABP, ECMO, PCI, and TTM. There is no standardized method for estimating the appropriate sample size for LCA.

Based on several simulation studies aimed at consistently high accuracy, a target sample size of > 500 was set. Statistical analyses were conducted using R software (R Foundation for Statistical Computing, Vienna, Austria), and statistical significance was set at *p* < 0.05.

## Results

### Study participants

**Fig. 1:**
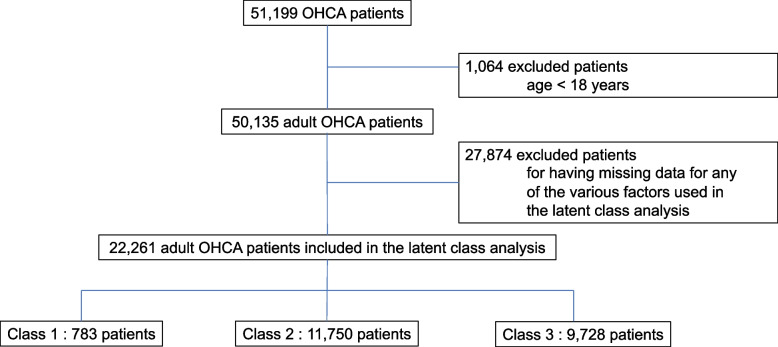
Flow of study participants and the number of participants in each sub-phenotype. OHCA, out-of-hospital cardiac arrest

Of the 51,199 patients who experienced OHCA and were included in the JAAM OHCA Registry, 1,064 were excluded as they were under the age of 18 years and 27,874 were excluded due to the lack of one of the various factors used in the LCA. Finally, 22,261 adult patients who experienced OHCA were included in the LCA (Fig. 1).

#### LCA

**Fig. 2:**
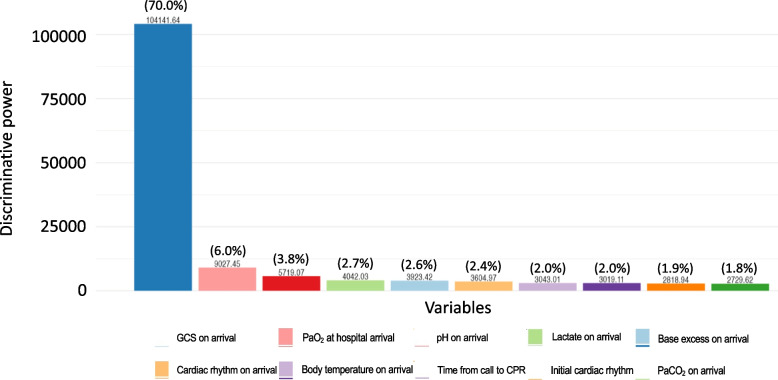
Discriminative power of each variable in descending order. GCS, Glasgow coma scale; PaCO_2_, partial pressure of carbon dioxide in arterial blood; PaO_2_, partial pressure of oxygen in arterial blood

Three sub-phenotypes were identified in the dataset of this study due to LCA. Patient backgrounds along with pre- and in-hospital characteristics in each latent class are listed in Table 1 and missing values are shown in e-Tables 1–3 in Supplementary file 1. The factor with the highest discriminative power upon patient’s arrival was the GCS score, followed by PaO_2_ (Fig. 2). The values obtained were as follows: Group 1, median (IQR) GCS: 7 (6–13), PaO_2_: 117.3 (64.0–241.7); Group 2, median (IQR) GCS: 3 (3–3), PaO_2_: 49.6 (19.5–120.0); and Group 3, median (IQR) GCS: 3 (3–3), PaO_2_: 29.1 (16.5–57.9).

**Table 1:** Pre-hospital and in-hospital characteristics in each latent class

**Variables**	**Group 1**	**Group 2**	**Group 3**	***P* value**
	**(*n* = 783)**	**(*n* = 11,750)**	**(*n* = 9,728)**	
**Pre-hospital variables**				
Age, median (IQR), year	69 (56–79)	76 (65–85)	75 (63–84)	<0.0001
Male, n (%)	563 (71.9)	7,290 (62.0)	5,740 (59.0)	<0.0001
Witness, n (%)				0.0005
EMS personnel	478 (61.0)	6,556 (55.8)	1570 (16.1)	
Others	201 (25.7)	807 (6.9)	1,104 (11.3)	
None	104 (13.3)	4,387 (37.3)	7,054 (72.5)	
Bystander CPR, n (%)	380 (48.5)	5,548 (47.2)	4,060 (41.7)	<0.0001
Initial cardiac rhythm monitored, n (%)				0.0005
Vf/pulseless VT	340 (43.4)	1,795 (15.3)	160 (1.6)	
PEA	231 (29.5)	4,392 (37.4)	1,264 (13.0)	
Asystole	57 (7.3)	5,146 (43.8)	7,797 (80.2)	
others	155 (19.8)	417 (3.5)	507 (5.2)	
Pre-hospital physician contact, n (%)	135 (17.2)	1,441 (12.3)	1,023 (10.5)	<0.0001
Pre-hospital shock delivery, n (%)	406 (51.9)	2,455 (20.9)	444 (4.6)	<0.0001
Pre-hospital adrenaline administration, n (%)	104 (13.3)	4,266 (36.3)	3,001 (30.8)	<0.0001
Pre-hospital advanced airway management, n (%)	192 (24.5)	7,114 (60.5)	5,568 (11.4)	<0.0001
Time from call to CPR, median (IQR), min	9 (7–12)	8 (7–10)	9 (8–13)	<0.0001
Time from call to hospital arrival, median (IQR), min	32 (26–40)	32 (26–38)	35 (29–44)	<0.0001
Origin of cardiac arrest, n (%)				0.0005
Acute coronary syndrome	271 (34.6)	988 (8.4)	279 (2.9)	
Cardiac origin	190 (24.3)	1,048 (8.9)	366 (3.8)	
Presumed cardiac origin	89 (11.4)	4,525 (38.5)	4,718 (48.5)	
Non-cardiac origin	233 (29.8)	5,189 (44.2)	4,365 (44.9)	
ROSC at hospital admission, n (%)	687 (87.7)	2,614 (22.2)	171 (1.8)	<0.0001
**In-hospital variables**				
Glasgow coma scale on arrival, median (IQR)	7 (6–13)	3 (3–3)	3 (3–3)	<0.0001
Body temperature on arrival, median (IQR), °C	36.0 (35.3–36.4)	35.8 (35.1–36.3)	35.5 (34.0–36.3)	<0.0001
Cardiac rhythm on arrival, n (%)				0.0005
Vf/pulseless VT	55 (7.0)	785 (6.7)	148 (1.5)	
PEA	47 (6.0)	3,155 (26.9)	1,325 (13.6)	
Asystole	32 (4.1)	5,412 (46.1)	8,113 (83.4)	
Others	649 (82.9)	2,398 (20.4)	142 (1.5)	
ECMO pump-on, n (%)	59 (7.5)	749 (6.4)	201 (2.1)	<0.0001
IABP, n (%)	103 (13.2)	647 (5.5)	86 (0.9)	<0.0001
Percutaneous coronary intervention, n (%)	211 (26.9)	596 (5.1)	71 (0.7)	<0.0001
Targeted temperature management, n (%)	243 (31.0)	1,267 (10.8)	189 (1.9)	<0.0001
pH at hospital arrival, median (IQR)	7.26 (7.12–7.34)	6.99 (6.90–7.10)	6.76 (6.66–6.985)	<0.0001
PaO_2_ at hospital arrival, median (IQR), mmHg	117.3 (64.0–241.7)	49.6 (19.5–120.0)	29.1 (16.5–57.9)	<0.0001
PaCO_2_ at hospital arrival, median (IQR), mmHg	41.6 (34.4–53.2)	75.3 (56.3–93.5)	102.2 (77.4–130.0)	<0.0001
Bicarbonate at hospital arrival, median (IQR), mEq/L	18.0 (14.7–21.1)	17.3 (13.7–20.8)	13.4 (10.0–16.4)	<0.0001
Base excess at hospital arrival, median (IQR), mmol/L	-8.3 (-13.8 – -4.7)	-14.2 (-18.2 – -9.8)	-23.0 (-27.0 – -19.0)	<0.0001
Serum lactate level at hospital arrival, median (IQR), mEq/L	63.9 (41.7–93.8)	96.3 (73.0–118.8)	153.0 (126.0–180.6)	<0.0001
Glucose level at hospital arrival, median (IQR), mg/dL	226 (178–289)	233 (149–309)	211 (113–321)	<0.0001
NH_3_ at hospital arrival, median (IQR),	59 (38–115)	172 (97–278)	436 (273–661)	<0.0001
PaO_2_ at 24 h from hospital arrival, median (IQR), mmHg	109.0 (85.7–132.3)	113.0 (88.9–151.1)	106.4 (82.0–145.0)	0.004
Bae excess at 24 h from hospital arrival, median (IQR), mmol/L	0.3 (-2.1–1.9)	-0.9 (-3.4–1.6)	-1.9 (-5.3–0.9)	<0.0001
Serum lactate level at 24 h from hospital arrival, median (IQR), mEq/L	11.7 (9.0–16.2)	16.2 (9.9–26.1)	18.9 (12.6–32.4)	<0.0001

*CPR* cardiopulmonary resuscitation, *ECMO* extracorporeal membrane oxygenation, *EMS* emergency medical services, *IABP* intra-aortic balloon pumping, *IQR* interquartile range, *PaCO*_*2*_ partial pressure of carbon dioxide in arterial blood, *PaO*_*2*_ partial pressure of oxygen in arterial blood, *PEA* pulseless electrical activity, *ROSC* return of spontaneous circulation, *SD* standard deviation, *Vf* ventricular fibrillation, *VT* ventricular tachycardia

### Sub-phenotypes and prognosis

Thirty-day survival with favorable neurological outcomes (CPC scores 1 and 2) as a primary outcome was evident in 66.0% participants belonging to Group 1, 5.2% in Group 2, and 0.5% in Group 3. Thirty-day survival as a secondary outcome was observed in 80.6% of the participants in Group 1, 11.8% in Group 2, and 1.3% in Group 3 (Table 2).

**Table 2:** Clinical outcomes of the study population in each latent class

**Variables**	**Group 1**	**Group 2**	**Group 3**	***P* value**
	**(*n* = 783)**	**(*n* = 11,750)**	**(*n* = 9,728)**	
30-day favorable neurological outcome, n (%)	517 (66.0)	611 (5.2)	53 (0.5)	<0.0001
30-day survival, n (%)	631 (80.6)	1,389 (11.8)	128 (1.3)	<0.0001
30-day cerebral performance category, n (%)				0.0005
1, good cerebral recovery	454 (54.2)	426 (3.6)	43 (0.4)	
2, moderate cerebral disability	93 (11.9)	185 (1.6)	10 (0.1)	
3, severe cerebral disability	75 (9.6)	237 (2.0)	27 (0.3)	
4, coma or vegetative state	39 (5.0)	541 (4.6)	48 (0.5)	
5, death or brain death	152 (19.4)	10,361 (88.2)	9,600 (98.7)	

### Logistic regression analysis

Logistic regression analysis after adjustment for covariates for 30-day survival with favorable neurological outcome revealed OR (95% CI) of 137.1 (99.4–192.2) in Group 1 and 4.59 (3.46–6.23) in Group 2 in comparison to Group 3. For 30-day survival, the OR (95%CI) was 161.7 (124.2–212.1) in Group 1 and 5.78 (4.78–7.04) in Group 2, compared with Group 3 (Supplementary e-Tables 4 and 5).

## Discussion

### Main findings

The pre-hospital clinical factors and factors available immediately after patient’s arrival at the hospital aided in classification of the three sub-phenotypes. Univariate analysis of neurological outcomes at 30 days showed 66.0% of the study participants belonging to sub-phenotype group 1, 5.2% to sub-phenotype group 2, and 0.5% to sub-phenotype group 3. The results of multivariate logistic regression analysis adjusted for diagnosis and treatment (cause of CPA, ECPR, IABP, PCI, and TTM) after admission also showed a similar trend. The most important factor contributing to sub-phenotype classification was the GCS score on admission, followed by PaO_2_ on admission.

### Association between GCS and prognosis

Previous studies on GCS on arrival have also reported an impact on neurological outcomes in patients who experienced OHCA [44, 45]. Nadolny et al. used multivariate logistic regression analysis in patients who experienced OHCA and achieved ROSC upon arrival and found that GCS > 4 was an independent predictor of in-hospital survival after OHCA (OR 6.4; 95% CI 2.0–20.3; *p* < 0.0001) [44]. Sondergaard et al. also reported that the 30-day survival for patients who experienced OHCA, achieved ROSC, and were conscious (GCS > 8) was higher than that for patients who attained ROSC but were comatose or hospitalized with ongoing CPR [45]. Among the patients in the current study, sub-phenotype group 1, with the most favorable neurological outcome, had a median (IQR) GCS of 7 (6–13) at arrival, whereas sub-phenotype group 3, with the worst neurological outcome, had a median (IQR) GCS of 3 (3–3), which was significantly different (*p* < 0.0001). A GCS score of 3 does not necessarily indicate that brain function has ceased; however, as in previous studies, a high GCS score suggests that brain function remains consistent with the possibility of a favorable neurological outcome.

### Association between PaO_2_ and prognosis

A high PaO_2_ on arrival indicates that circulation and respiratory control were well maintained during CPR and after ROSC and that blood flow to the major organs of the body, including the brain, is maintained. Adequate oxygenation of the brain tissue is an essential component of good neurological outcomes [46–48]. Several studies using near-infrared spectroscopy have shown that maintaining a balance between oxygen demand and supply in the brain surface tissues can predict neurological outcomes in patients who experienced OHCA [49, 50]. Therefore, it is theoretically consistent that maintaining PaO_2_ within normal limits is associated with favorable neurological outcomes in patients with OHCA. The relationship between arrival PaO_2_ and favorable neurological outcomes in this study was as follows: group 1 sub-phenotype, which had the best prognosis, had a median arrival PaO_2_ of 117.3 mmHg; group 2 sub-phenotype had a median arrival PaO_2_ of 49.6 mmHg; and group 3 sub-phenotype had the worst neurological outcome with a median arrival PaO_2_ of 29.1 mmHg. Patients with higher arrival PaO_2_ have better neurological outcomes. Consistent with our findings, previous studies have also reported an association of PaO_2_ with ROSC and subsequent survival [46–48]. The absence of extreme hyperoxemia has also been reported to be associated with favorable neurological outcomes, which is consistent with the findings of the present study [51, 52].

### Differences from previous studies

Two previous Japanese studies that identified the sub-phenotypes of OHCA differed from the present study in terms of the factors used for the identification of sub-phenotypes, which were associated with the neurological outcomes of OHCA [32, 33]. Three sub-phenotypes were identified in shockable rhythm, including PO_2_, PCO_2_, and cardiac rhythm upon arrival at the hospital, and the estimated glomerular filtration rate contributed to sub-phenotype identification [33]. In contrast, four sub-phenotypes were identified in the non-shockable rhythm, with PaO_2_, age, serum potassium, and estimated glomerular filtration rate contributing to sub-phenotype identification [32]. PaO_2_ was a common factor in all three OHCA sub-phenotype studies, including the present study, whereas other factors varied. Two previous studies used phenotypes restricted to the cardiac rhythm at the time of cardiac arrest. The present study used a phenotype that was not restricted to cardiac rhythm and included cardiac rhythm as a factor in the sub-phenotype classification. Cardiac rhythm (shockable or non-shockable) had no significant impact on sub-phenotype classification. Of the factors included in this study, the influence of initial cardiac rhythm was the sixth most important factor. Although the results of this study cannot be generalized to other populations, as different phenotypes may be identified and prognostic factors may differ depending on the set of phenotypes, we believe that it is important to include the effect of initial cardiac rhythm for the identification of the sub-phenotypes. Treatment decisions must be made immediately upon the arrival of patients at the hospital who experienced OHCA. In this study, we identified sub-phenotypes based on pre- and post-hospital factors that can be measured immediately upon arrival at the hospital, which would aid in making immediate treatment decisions. Factors that required a longer duration to obtain results, such as creatinine levels used in existing studies, were excluded from the analysis. This resulted in a time-oriented classification, and the prognostic factors were considered different from those in existing studies due to the different time ranges.

### The importance of basic life support and time lapse

Basic life support is extremely important for patients experiencing OHCA [53]. CPR is one of the most effective interventions and should be performed immediately after a cardiac arrest. Therefore, the time from call to CPR is important, because previous studies have reported that the earlier is the CPR started, the better are the neurological outcomes [54, 55]. However, in the present study, the time from call to CPR was similar for the sub-phenotypes with the best and worst neurological outcomes. We hypothesized that the main reason for the lack of difference in the time from the call to CPR in this study was the fact that we dealt with the data regarding time from the emergency call, not the time from the actual cardiac arrest to the emergency call. If the actual cardiac arrest time is longer, it can be inferred that a shorter duration from the call to CPR does not contribute to improved neurological outcomes. Factors related to CPR, such as the time from cardiac arrest to CPR, time from cardiac arrest to hospital arrival, and the presence of witnesses, may affect neurological outcomes.

### Clinical applications

Although it is possible to estimate the groups with a good prognosis even with clinical acumen, the classification of sub-phenotypes that contribute to differences in neurological outcomes in patients who experience OHCA has significant implications for treatment strategies. For patients with favorable neurological outcome factors, more intensive monitoring and therapeutic interventions may further improve the patient’s prognosis. However, for patients with poor neurological outcome factors, treatment discontinuation may help avoid futile treatment. Among the sub-phenotypes identified in this study, patients with higher GCS and PaO_2_ upon their arrival at the hospital had better neurological outcomes; otherwise, they were more likely to have poor neurological outcomes. Such information may influence decision-making regarding therapeutic interventions for patients with OHCA after they arrive at the hospital. As mentioned above, it may possible to estimate that GCS and PaO_2_ are related to prognosis with clinical acumen. However, recognition of these clinical data will be necessary for clinical practice and for future studies investigating prognostic factors. Since the data utilization rules of the OHCA registry did not allow for analysis other than the predefined tasks in this study, we intend to investigate the prognostic factor in future studies with clinically subdivided groups based on the clearly good prognosis (Group 1) and poor prognosis (Groups 2 and 3) phenotypes and the hypotheses proposed in this pilot study.

### Study limitations

This study has a few limitations. First, the number of factors used in the analysis may not have been sufficient. The factors used in the LCA in this study were extracted from the data in the Japanese OHCA Registry based on their clinical usefulness and importance. When different factors are included in LCA, different sub-phenotypes may be identified. Thus, there may be another sub-phenotype that predicts neurological outcomes more accurately. Second, the factors used in the LCA in this study were limited to pre-hospital factors and factors that could be measured immediately upon arrival at the hospital (i.e., factors that could be measured within a few minutes, such as blood gas findings). Factors that took longer to measure (e.g., blood tests and imaging findings) were not included in the factors used for sub-phenotype classification. This is because the aim of this study was to use sub-phenotype classification to determine whether invasive medical procedures such as Advanced Cardiovascular Life Support (ACLS) and ECPR should be performed in the Emergency Department soon after the patient arrives at the hospital. However, it might have been better to perform LCA by dividing patients according to the presence or absence of ROSC upon arrival at the hospital, because the treatment strategy, including ACLS, is likely to be different for patients who have ROSC upon arrival at the hospital and those who do not. Third, there were several missing data used in this study. The most common missing data were body temperature and blood gas analysis, both of which should be measured in the OHCA Registry. In cases where they were not measured, it is assumed that the condition was difficult to measure for unintentional and accidental reasons (e.g., understaffed conditions with other priorities for treatment). There was no consistent reason for the lack of measurement, and the missing data were assumed to be random and not imputed. Fourth, there is a wide range of clinical situations in which PaO_2_ is measured, and the physiological significance of PaO_2_ is likely to be different in patients with and without ROSC. Therefore, although PaO_2_ is an important factor, it should be considered that specific clinical situations and the lack of FiO_2_ can limit its interpretation. Fifth, this LCA cannot be applied immediately to clinical practice. However, it is possible to classify phenotypes with different prognoses using factors that are available immediately upon arrival at the hospital, and to have a model that can predict which phenotype a patient will have on presentation to the hospital. As in previous LCAs, no attempts have been made to create that model [24, 30]. The aim of this LCA was not the classification itself, but rather to classify the patients and homogenize the clinical heterogeneity to identify factors that influence prognosis since it is considered a guide for the generation of hypotheses to improve prognosis. Sixth, no validation was performed in this study; therefore, it remains unknown whether the identified sub-phenotypes would apply to other populations. Finally, the external validity of this study was limited. The criteria for transporting patients who experience OHCA vary widely among healthcare systems globally, and there is a high degree of heterogeneity in the context in which patients are transported to hospitals. Therefore, the sub-phenotypes identified in this study may have low external validity for countries with healthcare systems different from Japan; however, the results of this study can be extrapolated to regions where patients who experience OHCA are transported under healthcare systems similar to those in Japan.

## Conclusions

Three sub-phenotypes were identified in patients with OHCA by performing LCA of factors available immediately upon arrival at the hospital to predict neurological outcomes. The prognostic factors identified in this study may be useful in guiding the management of patients who suffer OHCA upon hospital arrival. However, these factors may have low external validity and require further investigation.

### Supplementary Information

Supplementary Material 1.

## Data Availability

Not applicable.

## Abbreviations

ACLS: Advanced cardiovascular life support
ARDS: Acute respiratory distress syndrome
CIs: Confidence intervals
CPA: Cardiopulmonary arrest
CPC: Cerebral performance category
CPR: Cardiopulmonary resuscitation
ECMO: Extracorporeal membrane oxygenation
ECPR: Extracorporeal membrane oxygenation-assisted cardiopulmonary resuscitation
GCS: Glasgow coma scale
IABP: Intra-aortic balloon pumping
IQR: Interquartile range
JAAM: Japanese Association for Acute Medicine
LCA: Latent class analysis
OHCA: Out-of-hospital cardiac arrest
ORs: Odds ratios
PaCO_2_: Partial pressure of carbon dioxide in arterial blood
PaO_2_: Partial pressure of oxygen in arterial blood
PCI: Percutaneous coronary intervention
ROSC: Return of spontaneous circulation
TTM: Targeted temperature management
