# Supplementary material for: Association between sub-phenotypes identified using latent class analysis and neurological outcomes in patients with out-of-hospital cardiac arrest in Japan
Source: BMC Cardiovasc Disord. 2024 Jun 14;24:303. doi: 10.1186/s12872-024-03975-z (PMC11177357; doi:10.1186/s12872-024-03975-z)
Supplement: Supplementary file 1 — Supplementary Material 1. [file 12872_2024_3975_MOESM1_ESM.docx]

Supplementary file 1

**Association between sub-phenotypes identified using latent class analysis and neurological outcomes in patients with out-of-hospital cardiac arrest in Japan**

Index

| e-Table 1 | Number of missing data of patient characteristics variables used for latent class analysis with analyzed patients |
| --- | --- |
| e-Table 2 | Number of missing data of patient characteristics and in-hospital treatment variables used for logistic regression analysis with analyzed patients |
| e-Table 3 | Number of missing data of in-hospital variables and clinical outcomes with analyzed patients |
| e-Table 4 | Logistic regression analysis after adjusted with covariates for 30-day favorable neurological outcomes and 30-day survival after cardiac arrest |
| e-Table 5 | Variance Inflation Factor (VIF) |

**e-Table 1. Number of missing data of patient characteristics variables used for latent class analysis with analyzed patients**

|  | **Overall** |
| --- | --- |
| **Variables** | **(n=50,135)** |
| **Pre-hospital variables** |  |
| Age, n (%) | 0 (0) |
| Male, n (%) | 0 (0) |
| Witness, n (%) | 0 (0) |
| Bystander CPR, n (%) | 0 (0) |
| Initial cardiac rhythm monitored, n (%) | 0 (0) |
| Pre-hospital physician contact, n (%) | 0 (0) |
| Pre-hospital shock delivery, n (%) | 0 (0) |
| Pre-hospital adrenaline administration, n (%) | 0 (0) |
| Pre-hospital advanced airway management, n (%) | 0 (0) |
| Time from call to CPR, n (%) | 818 (1.6) |
| Time from call to hospital arrival, n (%) | 181 (0.4) |
| ROSC at hospital admission, n (%) | 0 (0) |
| **In-hospital variables** |  |
| Glasgow coma scale on arrival, n (%) | 11 (0.02) |
| Body temperature on arrival, n (%) | 19,384 (38.7) |
| Cardiac rhythm on arrival, n (%) | 0 (0) |
| pH at hospital arrival, n (%) | 11,547 (23.0) |
| PaO_2_ at hospital arrival, n (%) | 12,333 (24.6) |
| PaCO_2_ at hospital arrival, n (%) | 11,836 (23.6) |
| Bicarbonate at hospital arrival, n (%) | 12,817 (25.6) |
| Base excess at hospital arrival, n (%) | 13,087 (26.1) |
| Serum lactate level at hospital arrival, n (%) | 12,360 (24.7) |

CPR, cardiopulmonary resuscitation; PaCO_2_, partial pressure of carbon dioxide in arterial blood; PaO_2_, partial pressure of oxygen in arterial blood; ROSC, return of spontaneous circulation

**e-Table 2. Number of missing data of patient characteristics and in-hospital treatment variables used for logistic regression analysis with analyzed patients**

|  | **Group 1** | **Group 2** | **Group 3** |
| --- | --- | --- | --- |
| **Variables** | **(n=783)** | **(n=11,750)** | **(n=9,728)** |
| **Pre-hospital variables** |  |  |  |
| Origin of cardiac arrest, n (%) | 0 (0) | 0 (0) | 0 (0) |
| **In-hospital variables** |  |  |  |
| ECMO pump-on, n (%) | 0 (0) | 0 (0) | 0 (0) |
| IABP, n (%) | 0 (0) | 0 (0) | 0 (0) |
| PCI, n (%) | 0 (0) | 0 (0) | 0 (0) |
| TTM, n (%) | 0 (0) | 0 (0) | 0 (0) |

ECMO, extracorporeal membrane oxygenation; IABP, intra-aortic balloon pumping; PCI, Percutaneous coronary intervention; TTM, Targeted temperature management

**e-Table 3. Number of missing data of in-hospital variables and clinical outcomes with analyzed patients**

|  | **Group 1** | **Group 2** | **Group 3** |
| --- | --- | --- | --- |
| **Variables** | **(n=783)** | **(n=11,750)** | **(n=9,728)** |
| **In-hospital variables** |  |  |  |
| Glucose level at hospital arrival, n (%) | 5 (0.6) | 122 (1.0) | 130 (1.3) |
| NH_3_ at hospital arrival, n (%) | 569 (72.7) | 8,708 (74.1) | 7,231 (74.3) |
| PaO_2_ at 24 hours from hospital arrival, n (%) | 459 (58.6) | 10,225 (87.0) | 9,463 (97.3) |
| Base excess at 24 hours from hospital arrival, n (%) | 462 (59.0) | 10,232 (87.1) | 9,464 (97.3) |
| Serum lactate level at 24 hours from hospital arrival, n (%) | 464 (59.3) | 10,263 (87.4) | 9,469 (97.3) |
| **Clinical outcomes** |  |  |  |
| 30-day favorable neurological outcome, n (%) | 0 (0.0) | 0 (0.0) | 0 (0.0) |
| 30-day survival, n (%) | 0 (0.0) | 0 (0.0) | 0 (0.0) |

PaO_2_, partial pressure of oxygen in arterial blood

**e-Table 4. Logistic regression analysis after adjusted with covariates for 30-day favorable neurological outcomes and 30-day survival after cardiac arrest**

|  | **Odds ratio** | **95% CI** |
| --- | --- | --- |
| **For 30-day neurologically favorable outcome** |  |  |
| Complete case analysis |  |  |
| Group 1 | 137.1 | 99.4-192.2 |
| Group 2 | 4.59 | 3.46-6.23 |
| Group 3 | ref | ref |
|  |  |  |
| **For 30-day survival** |  |  |
| Complete case analysis |  |  |
| Group 1 | 161.7 | 124.2-212.1 |
| Group 2 | 5.78 | 4.78-7.04 |
| Group 3 | ref | ref |

CI, confidence interval; OR, odds ratio

**e-Table 5. Variance Inflation Factor (VIF)**

|  | 30-day favorable neurological outcome | 30-day survival |
| --- | --- | --- |
| Origin of cardiac arrest | 2.18 | 2.16 |
| ECMO pump-on | 1.58 | 1.92 |
| IABP | 1.95 | 2.28 |
| PCI | 2.32 | 2.34 |
| TTM | 1.37 | 1.32 |

ECMO, extracorporeal membrane oxygenation; IABP, intra-aortic balloon pumping; PCI, Percutaneous coronary intervention; TTM, Targeted temperature management
